# A match made in health care: can ethics and governance better support impactful implementation research?

**DOI:** 10.5694/mja2.70109

**Published:** 2026-01-18

**Authors:** Natalie Taylor, Zhicheng Li, Cathelijne van Kemenade, Jackie Curtis, Patrick Bolton

**Affiliations:** ^1^ University of New South Wales Sydney NSW; ^2^ The Sax Institute Sydney NSW

Researchers are increasingly required to demonstrate the impact of their work. In contrast to traditional academic measures such as publications and citations, impact is determined by the successful translation and implementation of research findings into real‐world settings that deliver direct benefits.^1^ Translating research into policy and practice is a complex and often difficult process. A key challenge is effectively and sustainably implementing evidence‐based interventions in the real world. Aside from controlled drug trials, making an impact in health care — such as driving behavioural or systemic change — and providing clear evidence of that impact, particularly demonstrating its sustainment, is regarded as the Holy Grail.^2^ Applied research in health care often fails to demonstrate a meaningful contribution to health outcomes.^3^, ^4^

## Research ethics and governance: bureaucratic hurdles or opportunities?

When research fails to deliver impactful outcomes, research ethics and governance (REG) processes are often scrutinised for causing unnecessary delays and burdens (Box 1 shows the REG process in Australia for a multisite research application and the behind‐the‐scenes work). REG reviews are crucial in safeguarding the health and wellbeing of research participants and ensuring the compliance of the research activities with relevant institutional, jurisdictional and national standards. However, ineffective and bureaucratic regulation can lead to hyper‐regulation, where the regulation, governance and management become excessively burdensome and disproportionate to the conceivable risks the research poses to participants.^2^, ^5^ This is particularly challenging for implementation studies, which are at the nexus of clinical research and impact. This positioning introduces the challenge of defining the unique contribution to health outcomes within a research project and distinguishing its immediate role from the broader, long term effects the research may have on widespread change, which can take years or even decades to achieve. Although REG delays can compound these challenges, we argue that they also present opportunities to build meaningful relationships, enhance health systems research, and support lasting impact.

Box 1 **Process map of the research ethics and governance processes in Australia**

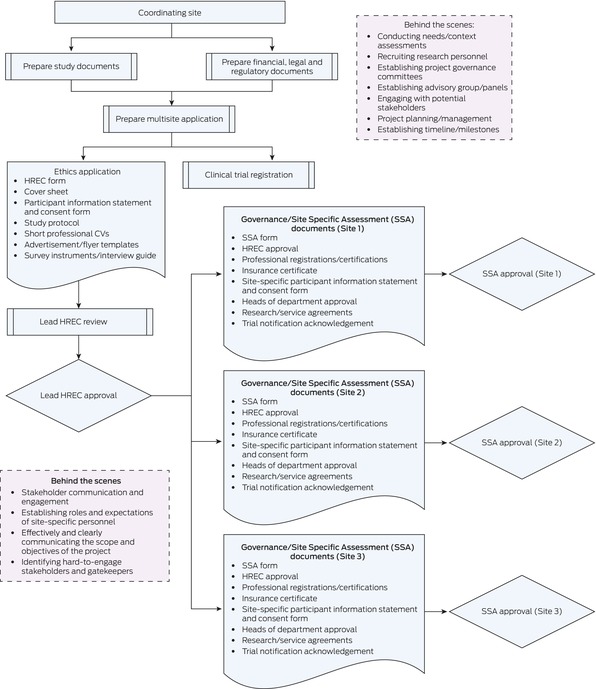

HREC = human research ethics committee; SSA = site‐specific assessment.

This article reflects on two implementation experiences in Australian hospital settings and presents an evidence‐driven approach for categorising implementation barriers and identifying strategies to leverage REG processes as opportunities. Barriers encountered in these two case studies, directly and indirectly related to REG, were identified to highlight lessons learned from our successes and setbacks. To help overcome these barriers, we mapped them against two well‐known implementation science frameworks: the Consolidated Framework for Implementation Research (CFIR) and the Expert Recommendations for Implementation Change (ERIC) implementation strategies (Box 2).^6^, ^7^ Operationalising these theory‐driven strategies within the context of Australian health care and REG is crucial for achieving research impact and fostering sustained change.

Box 2 **Strategies for addressing regulatory barriers for implementation research**

**Table:** 

Barriers	HaSP and S3L experiences	CFIR domain (subdomain)	ERIC strategies
Barrier 1: The lengthy bureaucratic processes and administrative burden of obtaining relevant research governance approvals	HaSP and S3L identified health care workers from within the hospital networks as implementation leads (IL) to drive an implementation effort internally. The recruitment resulted in the need for a Services Agreement between each hospital health district and the research organisation, which was interdependent with REG	Outer setting (Policies and laws; Local conditions)	Build a coalition* Capture and share local knowledge Identify and prepare champions Conduct local consensus discussions
Barrier 2: The REG framework is not compatible with the nature of implementation studies, which generally focus on interventions that have already been proven to be safe and effective	The challenge of following the rigid, highly controlled REG framework and protocol specifications that had been designed for clinical and drug interventions. For example, the Good Clinical Practice guidelines were less relevant for HaSP and S3L	Outer setting (Policies and laws)	Build a coalition Capture and share local knowledge Identify and prepare champions
Barrier 3: The clinical trial classification overestimates the risk of implementation studies	Health service interest holders were reluctant to actively engage and support the implementation of HaSP and S3L, compounded by gatekeeping from authorising personnel within the hospital networks	Individuals (Knowledge and beliefs about the intervention) Inner setting (Relational connections; Communications)	Promote network weaving Build a coalition Create a learning collaborative Conduct educational meetings/outreach visits Identify and prepare champions Assess for readiness and identify barriers and facilitators
Barrier 4: Unclear roles and responsibilities among health service interest holders	The expectation from health service interest holders for S3L was that the researchers would “come in” and “fix the smoking problem”, which showed a gap in communication regarding the study objectives, roles and responsibilities	Inner setting (Incentive systems) Individuals (Motivation) Implementation process (Engaging innovation deliverers)	Audit and provide feedback Identify and prepare champions Assess for readiness and identify barriers and facilitators Create a learning collaborative Involve executive boards
Barrier 5: Lack of leadership support from middle management	The research team of S3L struggled to establish crucial relationships with middle managers to engage an already busy clinical workforce	Inner setting (Relational connections) Individuals (Leaders)	Promote network weaving Build a coalition Create a learning collaborative Involve executive boards Identify and prepare champions Obtain formal commitments
Barrier 6: Difficulties in effectively communicating the study rationale and importance of implementation research to health service interest holders	Despite having ILs to lead the implementation internally for HaSP and S3L, there was a general lack of understanding of implementation studies among the health service interest holders	Inner setting (Communication; Relative priority)	Promote network weaving Build a coalition Create a learning collaborative Assess for readiness and identify barriers and facilitators Conduct local needs assessment Conduct local consensus discussions

CFIR = Consolidated Framework for Implementation Research; ERIC = Expert Recommendations for Implementation Change; HaSP = Hide and Seek Project; IL = implementation lead; REG = research ethics and governance; S3L = Stop Smoking Start Living study. *See the Supporting Information for the definitions of ERIC strategies.

## Implementation barriers: the Hide and Seek Project (HaSP) and the Stop Smoking Start Living (S3L) study

The HaSP and S3L study were two large scale, multisite, non‐pharmacological implementation trials aimed to implement health care interventions into routine practice across Australian hospital networks.^8^, ^9^, ^10^ The HaSP focused on improving detection of Lynch syndrome and the S3L study aimed to implement hospital‐based smoking cessation interventions.

Three key barriers were identified in the REG processes, including delays in the studies’ rollout and overall timeline due to the lengthy bureaucratic processes and administrative burden of obtaining relevant research governance approvals (Barrier 1), the incompatibility of the REG framework with the nature of implementation studies (Barrier 2), and an overestimation of risk of implementation studies under the clinical trial classification (Barrier 3).

Additional barriers included unclear roles and responsibilities among health service interest holders (Barrier 4), a lack of leadership support from middle management (eg, ward managers, nursing unit managers) (Barrier 5), and difficulties in effectively communicating the study rationale and importance of implementation practice and research to relevant clinical staff (Barrier 6). These barriers, although not directly related to the REG processes, collectively hindered interest holder engagement and diminished the overall momentum of implementation effort.

## Leveraging REG for implementation research: the application of CFIR–ERIC strategies

Mapping these barriers to CFIR–ERIC strategies highlighted opportunities to leverage the REG processes for greater impact. The following discussion explores ways to incorporate effective CFIR–ERIC implementation strategies into the REG processes to overcome barriers within the local context, establish a shared understanding among the key interest holders, and build relationships beyond academic collaborations.

### Overcoming barriers within the local context

In both the HaSP and the S3L study, a locally employed health care professional was appointed at 0.2 full‐time equivalent and trained as an implementation lead (IL) to coordinate the implementation (ERIC strategy: Identify and prepare champions). ILs were appointed through a formal application process to ensure that they had the relevant qualifications for the role as well as the soft skills for the behind‐the‐scenes work shown in Box 1.^11^ These soft skills included high level interpersonal and communication skills, ability to work collaboratively as part of a team, relationship‐building with external interest holders, and the ability to adapt and manage uncertainties. In addition, training was provided to ILs to develop foundational implementation science methodologies and practical skills, such as facilitating focus‐group consultations, identifying barriers to behaviour change, and maintaining interest holder engagement.^11^

In addition to formalising IL positions and implementation training, ILs should be supported from the outset to meet with local principal investigators to work through specific nuances for obtaining access to data and resources (ERIC strategies: capture and share local knowledge; conduct outreach visits; assess for readiness and identify barriers and facilitators). This process also offers an opportunity to understand the local context of the planned implementation and develop targeted strategies to mitigate and reduce the impact of REG delays during the implementation process.

The local context includes specific factors that must be considered in the implementation setting, such as environmental characteristics, organisational culture and values (eg, leadership structure, openness to new ideas), information technology and infrastructure (eg, electronic documentation systems, workforce composition), and the availability of resources and local support.^6^ By navigating the REG requirements, researchers (supported by the ILs) can identify early any potential factors within the local context that may positively or negatively impact implementation efforts. For implementation trials, understanding the context and maintaining flexibility in the methodological approach is as crucial, if not more important than, strictly adhering to a fixed research design.^1^

### Establish a shared understanding among the interest holders

The completion of HaSP resulted in over 50 theory‐informed implementation strategies proposed across seven hospitals.^9^ This success was largely due to effective engagement with health service interest holders (ERIC strategy: promote network weaving), particularly the collaborative relationships fostered by working through the REG processes together (ERIC strategies: build a coalition; create a learning collaborative) and using a pragmatic and flexible approach that addressed the nuances of the local context at the participating hospital (ERIC strategy: capture and share local knowledge). Our experience of the S3L study suggested that having the regulatory approvals and the sign‐off from hospital executives did not automatically generate support or engagement from mid‐level managers or the health care professionals on the ground. Collaboration on REG processes, as was undertaken with the HaSP, took time, but it fostered a shared understanding of the project goals and facilitated a joint approach to finding solutions to overcome the challenges.^12^

Box 3 presents an example of what can potentially drive the actions of researchers, health service interest holders, and REG officers to endorse and engage in research activities. A research project might achieve immediate commitment from health care interest holders if it meets the REG and clinical requirements. For longer term endorsement, researchers need to think beyond the project design process undertaken as part of a research grant submission to focus on how the impact of the research aligns with the interest of the different interest holders. REG processes provide an opportunity to discuss the research objectives and reach a level of local consensus on the importance of the research and the appropriateness of the clinical innovation among the various interest holders involved and obtain formal commitments from key partners to the proposed research and implementation activities. It is crucial to identify individuals who may be reluctant or face significant barriers during implementation and tailor communication strategies to negotiate and resolve any differences in their vested interests that may not completely align with the research objectives.^13^ It is possible to find a mutually beneficial middle ground and convert the potential gatekeepers into knowledge brokers through learning collaborative and effective interest holder engagement strategies.

Box 3 **Examples of incentives for engaging in implementation research from the perspectives of different interest holders in health**

**Table:** 

Interest holders	Short term incentives	Longer term incentives
Researchers	Academic achievements and recognition among peers, such as publications and conference presentations	Advance knowledge and generate impact; contribute to meaningful societal impact
Health care interest holders	Clinical requirement such as accreditation; professional development activities	Improve quality and safety of patient care
Ethics and governance regulators	Protect the welfare and rights of research participants and safeguard the public	Improve and maintain the quality of research

### Sustaining relationships beyond academic collaboration

“Successful implementation research begins and ends with successful collaboration.”^1^ This means working with interest holders who will be affected by the intervention. This can be achieved by promoting network weaving (ie, identify and build on existing high quality working relationships and networks within and outside the organisation) with frontline health care professionals delivering the intervention and patients receiving the intervention, as well as individuals involved before, during and after the implementation (eg, REG officers, hospital executives and managers, policy and law makers, advocacy groups). Interest holder engagement is increasingly recognised as crucial, yet it is also acknowledged as one of the most challenging aspects of health care and health service implementation.^1^, ^14^

There are tools to support engagement that can be used alongside implementation methods. For example, the Implementation‐STakeholder Engagement (I‐STEM) is a sensitising tool developed to be used alongside implementation frameworks to support interest holder engagement activities in health and social care implementation efforts.^15^ It focuses on the key processes for planning (identifying and prioritising engagement objectives and mapping interest holders using predefined criteria), engaging (defining qualities and logistics for the selected engagement approach), and evaluating engagement outcomes. Other strategies to maintain interest holder engagement in implementation research include transparent communication and regular updates on progress, establishing feedback mechanisms that allow interest holders to voice their opinions and concerns, developing tailored engagement strategies that consider the unique dynamics and needs of the interest holders, and obtaining leadership support from both the executive and the mid‐level managers.

## Call to action

Collectively, we need to rethink and reform the systems that govern the translation of evidence into practice through the application of evidence‐based, theory‐driven implementation strategies, supported by strong collaboration among researchers, health care interest holders, and REG officers. Researchers should work closely with health care interest holders and REG officers and advocate for dedicated resources that support the invisible work of implementation — relationship‐building, interest holder engagement, and iterative adaptation.^16^ Health care interest holders are powerful advocates for change; they can help to align clinical priorities with research goals, champion the integration of research into clinical workflows, and advocate for context‐sensitive implementation. REG officers should support ways to enhance the compatibility of implementation studies in the REG processes, incentivise collaboration across sectors, and allocate resources to sustain long term change. Investing in infrastructure that supports both implementation practice and research is critical to achieving real‐world impact.

## Conclusion

Achieving impact in health care is the goal of implementation research, yet it often remains elusive. In this article, we shared two real‐world cases of implementation research, offering valuable insights into the complexities of translating research into practice. By reflecting on these experiences, we identified key opportunities to integrate theory‐driven strategies within REG processes that can help overcome regulatory challenges and bolster opportunities for collaboration that leads to the desired research impact. However, leveraging these processes alone is not enough — broader complexities still hinder implementation success, and the scarcity of funding often forces us to overlook critical foundation steps. Moving forward, it is crucial that we rethink how we approach these challenges, optimising the existing infrastructure while advocating for resources to support the dogged behind‐the‐scenes efforts that drive knowledge translation and sustainable change.

### Ethics approval statement

The Stop Smoking Start Living study was approved by the South Eastern Sydney Local Health District human research ethics committee (2022/ETH01452); the Hide and Seek Project study was approved by the Royal Prince Alfred human research ethics committee (HREC/17/RPAH/542).

## Competing interests

No relevant disclosures.

## Provenance

Not commissioned; externally peer reviewed.

## Author contribution statement

Taylor N: Conceptualization, writing (original draft), approval of final version. Li Z: Conceptualization, writing (original draft), approval of final version. Van Kemenade C: Conceptualization, writing (review and editing), approval of final version. Curtis J: Conceptualization, writing (review and editing), approval of final version. Bolton P: Conceptualization, writing (review and editing), approval of final version.

## Supporting information


Supplementary tables
