# Supplementary material for: A match made in health care: can ethics and governance better support impactful implementation research?
Source: Med J Aust. 2026 Jan 18;224(1):e70109. doi: 10.5694/mja2.70109 (PMC12813301; doi:10.5694/mja2.70109)
Supplement: Supplementary file 1 — Supplementary tables [file MJA2-224-0-s001.pdf]

Supporting Information

Supplementary material

This appendix was part of the submitted manuscript and has been peer reviewed. It is posted as supplied by the authors.

Appendix to: Taylor N, Li Z, van Kemenade C, et al. A match made in health care: can ethics and governance better support impactful implementation research? *Med J Aust* 2025; doi: 10.5694/mja2.70109.

Expert Recommendations for Implementation Change (ERIC) strategies definitions

Build a coalition	Recruit and cultivate relationships with partners in the implementation effort
Capture and share local knowledge	Capture local knowledge from implementation sites on how implementers and clinicians made something work in their setting and then share it with other sites
Identify and prepare champions	Identify and prepare dedicated individuals to support, market, and drive through an implementation, overcome indifference or resistance that the intervention may provoke in an organisation
Conduct local consensus discussions	Include local providers and other stakeholders in discussions that address whether the chosen problem is important and whether the clinical innovation to address it is appropriate
Promote network weaving	Identify and build on existing high quality working relationships and networks within and outside the organisation to promote information sharing, collaborative problem-solving, and a shared vision/goal related to implementing the innovation
Create a learning collaborative	Facilitate the formation of groups of providers or provider organisations and foster a collaborative learning environment to improve implementation of the clinical innovation
Conduct educational meetings/outreach visits	Have a trained person meet with providers in their practice settings to educate providers about the clinical innovation with the intent of changing the provider's practice
Assess for readiness and identify barriers and facilitators	Assess various aspects of an organisation to determine its degree of readiness to implement, barriers that may impede implementation, and strengths that can be used in the implementation effort
Audit and provide feedback	Collect and summarise clinical performance data over a specified time period and give it to clinicians and administrators to monitor, evaluate, and modify provider behaviour
Involve executive boards	Involve existing governing structures (eg, boards of directors, medical staff boards of governance) in the implementation effort, including the review of data on implementation processes
Obtain formal commitments	Obtain written commitments from key partners that state what they will do to implement the innovation
Conduct local needs assessment	Collect and analyse data related to the need for innovation
